# Dietary Iron Intake and Mental and Behavioral Disorders Due to Use of Tobacco: A UK Biobank Study

**DOI:** 10.3390/nu17010039

**Published:** 2024-12-26

**Authors:** Xueting Qi, Ronghui Zhang, Hailong Zhu, Jia Luo, Qiuge Zhang, Weijing Wang, Tong Wang, Dongfeng Zhang

**Affiliations:** 1 Department of Epidemiology and Health Statistics, School of Public Health, Qingdao University, 308 Ningxia Road, Qingdao 266071, China; qixueting@qdu.edu.cn (X.Q.); 2021010151@qdu.edu.cn (R.Z.); luojia@qdu.edu.cn (J.L.); zhangqiuge@qdu.edu.cn (Q.Z.); wangwj@qdu.edu.cn (W.W.); zhangdongfeng@qdu.edu.cn (D.Z.); 2 Department of Operations Management, Qingdao Municipal Center for Disease Control and Prevention, Qingdao 266033, China; zhuhailong_1986@163.com

**Keywords:** dietary iron, tobacco, mental and behavioral disorders, UK Biobank

## Abstract

Background: Over 1 billion smokers worldwide, one-third of whom have mental and behavioral disorders, exist. However, factors influencing mental and behavioral disorders due to the use of tobacco remain largely unexplored. This study aims to investigate the relationship between dietary iron intake and mental and behavioral disorders due to the use of tobacco. Methods: Using large population cohort data from the UK Biobank (500,000 participants at 22 assessment centers between 2006 and 2010), we employed logistic and Cox regression analyses to explore both cross-sectional and longitudinal associations between dietary iron intake and mental and behavioral disorders due to the use of tobacco. Additionally, we assessed the nonlinear relationship between dietary iron intake and these disorders using restricted cubic spline plots. Results: Logistic regression analysis indicated that dietary iron intake was negatively associated with mental and behavioral disorders due to the use of tobacco. The Cox regression results supported a protective effect of increased dietary iron intake against these disorders. Stratified and sensitivity analyses were consistent with the primary findings. Restricted cubic spline plots revealed a nonlinear relationship between dietary iron intake and mental and behavioral disorders due to the use of tobacco. In the total sample, as well as in both age groups and the male subgroup, the risk reduction rate initially accelerated before slowing down. In contrast, the risk reduction rate in the female group declined rapidly at first and then leveled off. Conclusions: This study demonstrates that dietary iron intake has a protective effect against mental and behavioral disorders due to the use of tobacco, revealing a nonlinear association between these two traits. These findings provide important insights for the profilaxy and treatment of mental and behavioral disorders due to the use of tobacco in the future.

## 1. Introduction

The tobacco epidemic is one of the greatest public health threats that the world has ever faced [1]. The clear realization that tobacco is a risk factor for a variety of diseases, including lung cancer, attracts the vast majority of attention [2,3,4,5,6]. However, mental and behavioral disorders due to the use of tobacco have rarely been studied. Mental and behavioral disorders due to the use of tobacco refer to dependence syndrome, harmful use, withdrawal state, and acute intoxication, etc. [7]. These disorders are characterized by strong urges to smoke, the inability to control cessation, and withdrawal symptoms such as frustration, anxiety, and irritability [8]. There were more than 1 billion smokers worldwide in 2019, which is still increasing today [9]. According to the American Lung Association, 35% of smokers have behavioral health disorders [10]. Among those hospitalized for schizophrenia, depression, or bipolar disorder, approximately half of the deaths are due to smoking-related causes [11]. Therefore, it is critical to identify and manage the influence factors for mental and behavioral disorders due to the use of tobacco.

Tobacco use causes nicotine to bind to and activate acetylcholine receptors in the body, which results in dopamine release [12]. However, repeated exposure to nicotine leads to the desensitization of these receptors, resulting in decreased dopamine release and contributing to mental and behavioral disorders [13]. Iron, an essential nutrient, is closely related to physical and mental health [14,15,16,17,18]. One of the physiological roles of iron is to promote dopaminergic signaling by enhancing the expression of the D2 neurotransmitter, increasing dopamine release [19]. Based on this, we hypothesize that there is an association between dietary iron intake with mental and behavioral disorders due to the use of tobacco.

Many studies have currently reported connections between iron and mental and behavioral disorders, with inconsistent conclusions. For example, one study provided evidence of a negative correlation between circulating iron levels and depression and anxiety across different age and gender groups [20]. Similarly, another study found that dietary iron intake had a negative association with the risk of depression [21]. In contrast, one study indicated that excessive iron increased the risk of depression and anxiety [22]. Despite numerous investigations in the general population, there has been no research on the relationship between dietary iron intake and mental and behavioral disorders due to the use of tobacco among smokers [23,24].

Therefore, we conducted a cross-sectional and cohort study using data from the UK Biobank (UKB) to explore both the cross-sectional and longitudinal associations between dietary iron intake and mental and behavioral disorders due to the use of tobacco. Additionally, we employed restricted cubic spline curves to clarify the dose–response relationship between dietary iron intake and mental and behavioral disorders due to the use of tobacco.

## 2. Materials and Methods

### 2.1. Study Participant and Design

**Figure 1:**
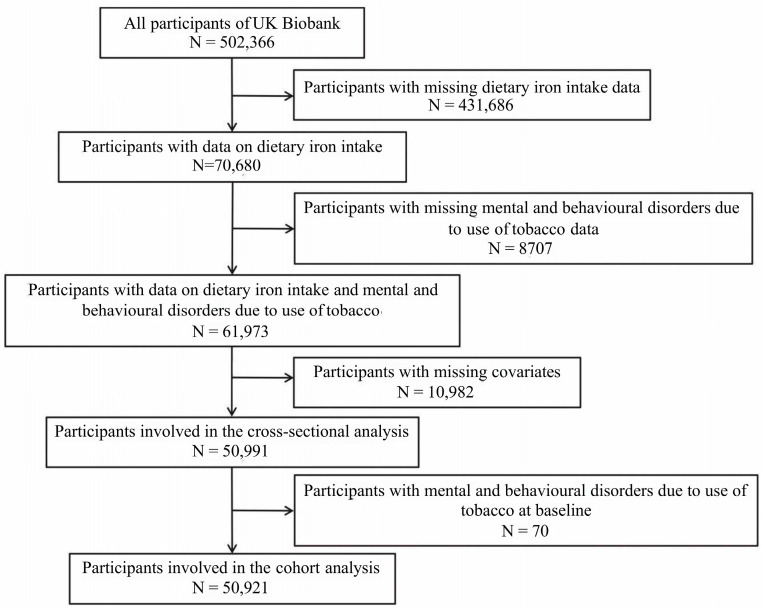
Flow diagram of study participant selection.

The UKB is a large prospective population-based study that assessed approximately 500,000 participants at 22 assessment centers between 2006 and 2010, including detailed health data, genomic information, and lifestyle data for each individual. These assessment centers covered various settings in the UK, ensuring a broad distribution of all exposures to detect reliable generalized associations between baseline characteristics and health outcomes. Additionally, the UKB was combined with UK national datasets for a longitudinal follow-up of individual information. The study has received ethical approval from the North West Multi-Centre Research Ethics Committee in the UK (reference number: 11/NW/0382, 16/NW10274, 21/NW/0157), and all participants provided written informed consent. The specific filtering process is illustrated in Figure 1. A total of 50,991 participants were included in the cross-sectional analysis. In the longitudinal analysis, the sample size was 50,921.

### 2.2. Exposure

Between April 2009 and September 2010, participants’ overall dietary intake information for the past 24 h was collected at assessment centers in the UK using an online questionnaire called Oxford WebQ. This 24 h dietary assessment tool is specifically designed for large prospective studies. It asks participants to provide dietary information using specified standard portion sizes within given food categories [25]. Statistical data indicated that Oxford WebQ correlated with most nutrients equal to or greater than interviewer-administered dietary information [26]. In the Oxford WebQ, the intake of food nutrients, including iron, was automatically calculated by multiplying the consumed portions by the predetermined quantity of each food and its corresponding nutrient content [27].

### 2.3. Outcome

The outcome of this study was mental and behavioral disorders due to the use of tobacco, identified using the International Classification of Diseases, 10th edition (ICD-10) codes. The outcome code was F17 in ICD-10, including F17.0 (acute intoxication), F17.1 (harmful use), F17.2 (dependence syndrome), F17.3 (withdrawal state), F17.4 (withdrawal state with delirium), F17.7 (residual and late-onset psychotic disorder), and F17.9 (unspecified mental and behavioral disorder), with more details available on the UKB website (https://www.ukbiobank.ac.uk/, accessed on 1 December 2024). New cases and the onset time were determined based on the first diagnosis recorded in the hospital inpatient database during the follow-up period. The date of death was obtained from the UK National Death Register. The follow-up period ended on the date of onset, date of death, date of loss to follow-up, or the date of observation termination (31 December 2022), with the earliest occurrence taking precedence.

### 2.4. Covariates

Based on previous research, the following factors were included as covariates in the study: age, sex (male, female), ethnicity (White, other), body mass index (BMI), physical activity level (low, moderate, high), energy intake, Townsend Deprivation Index (TDI), education qualifications (“college or university degree and other professional qualifications” were categorized as “College and above,” while the remaining qualifications were categorized as “Below College”), alcohol consumption (never, previous, current), employment status (“employed in paid employment or self-employed” was categorized as “Full-time work”, while the remaining categories were classified as “Non-full-time work”), hypertension (yes, no), stroke (yes, no), and diabetes (yes, no) [28,29,30,31,32,33,34,35,36]. Physical activity levels were classified based on Metabolic Equivalent Task (MET) score data derived from the International Physical Activity Questionnaire (IPAQ) guidelines [37].

### 2.5. Statistical Analysis

In descriptive studies, continuous variables were described by mean and standard deviation (SD) and categorical variables by numbers (N) and percentages (%). Dietary iron intake was divided into four quartiles (Q1: <25th percentile, Q2: 25th to 50th percentile, Q3: 50th to 75th percentile, and Q4: >75th percentile), with the lowest quartile as the reference group.

In cross-sectional studies, logistic regression models were employed to estimate the association between dietary iron intake and mental and behavioral disorders due to the use of tobacco. The results were reported as odds ratios (ORs) and 95% confidence intervals (CI).

In the cohort study, the Cox proportional hazards model was used to assess the longitudinal associations between dietary iron intake and mental and behavioral disorders due to the use of tobacco. The timescale was the follow-up duration from baseline to the occurrence of outcomes, death, loss to follow-up, or the end of observation (whichever occurred first, measured in days). The results were reported as hazard ratios (HRs) with 95% CI.

A total of four models were fitted in this study. Model 1 included no covariates. Model 2 adjusted for age, sex, ethnicity, education qualifications, employment status, and TDI. Model 3 further adjusted for BMI, physical activity level, energy intake, and alcohol consumption. Model 4 was additionally adjusted for hypertension, diabetes, and stroke.

To further evaluate the robustness of the cohort analysis results, we conducted the following sensitivity analyses: (1) Considering the chronic and long-term nature of the effects of dietary factors on mental and behavioral disorders in the cohort analysis, we excluded participants diagnosed with mental and behavioral disorders due to the use of tobacco in the 2 years prior to follow-up. (2) Given the strong association between sleep and mental health, we adjusted for sleep duration [38]. A sleep duration of 7–8 h was classified as normal sleep, while other durations were classified as abnormal sleep. (3) Participants with extreme iron intake or extreme energy intake were excluded (intake < first percentile and > ninety-ninth percentile). (4) Participants with a history of hypertension, diabetes, or stroke at baseline were excluded. (5) Iron supplements are adjusted to take into account that some individuals may use iron supplements. (6) We re-performed the primary analysis by dividing dietary iron intake into heme iron and non-heme iron. Moreover, stratified analyses were performed according to sex, age, and BMI, while the interaction was examined using a Wald test. Age was divided into two groups: ≤60 years and >60 years, and BMI was divided into four groups: <18.5 kg/m^2^, ≥18.5 kg/m^2^ and <25 kg/m^2^, ≥25 kg/m^2^ and <30 kg/m^2^, and ≥30 kg/m^2^.

Additionally, we treated dietary iron intake as a continuous variable and employed restricted cubic splines to examine the dose–response relationship between dietary iron intake and mental and behavioral disorders due to the use of tobacco. Considering the varying iron requirements based on age and sex, we investigated the dose–response relationships separately for different gender and age groups.

Statistical analyses were performed in R version 4.2.3, and a *p*-value < 0.05 for a two-sided test was considered statistically significant.

## 3. Results

### 3.1. Participant Baseline Characteristics

The baseline characteristics of study participants grouped by dietary iron intake are shown in Table 1. Compared to participants with the lowest dietary iron intake, those with the highest intake were more likely to be male, non-full-time workers, and individuals with diabetes. They tended to have a lower BMI and TDI while exhibiting higher levels of physical activity, energy intake, education qualifications, and alcohol consumption.

### 3.2. Cross-Sectional Analysis

At baseline, there were 70 patients with mental and behavioral disorders due to the use of tobacco. The results of the cross-sectional analysis are displayed in Table 2. According to the results, all four models showed that dietary iron intake was negatively associated with mental and behavioral disorders due to the use of tobacco. Model 4 (adjusted for age, sex, ethnicity, BMI, physical activity level, energy intake, TDI, education qualifications, alcohol consumption, employment status, hypertension, stroke, and diabetes) indicated that compared to the Q1 group, the ORs (95% CI) for the Q2, Q3, and Q4 groups were 0.42 (0.21–0.83), 0.26 (0.12–0.60), and 0.41 (0.18–0.98), respectively.

### 3.3. Cohort Analysis

During a median follow-up of 6.84 years, 1404 new cases of mental and behavioral disorders due to the use of tobacco were identified. The association between dietary iron intake and the risk of mental and behavioral disorders due to the use of tobacco is presented in Table 3. The Cox proportional hazards model did not violate the proportional hazards assumption. According to the results, Model 4 indicated that the risk of mental and behavioral disorders due to the use of tobacco was reduced in the Q2 (HR: 0.60, 95% CI: 0.53–0.68, *p*: <0.001), Q3 (HR: 0.59, 95% CI: 0.52–0.67, *p*: <0.001), and Q4 (HR: 0.50, 95% CI: 0.43–0.58, *p*: <0.001) groups compared to the Q1 group, and this association was observed in Models 1, 2, and 3.

### 3.4. Stratified Analysis

The stratified analysis results are shown in Supplementary Table S1. When stratified by age, both age groups resulted in a reduced risk of mental and behavioral disorders due to the use of tobacco as dietary iron intake increased. In the ≤60 age group, the HR (95% CI) was 0.58 (0.50–0.67), 0.57 (0.49–0.67), and 0.45 (0.37–0.55) for the Q2, Q3, and Q4 groups, respectively, compared to the Q1 group. In the >60 age group, the HR (95% CI) was 0.62 (0.51–0.76), 0.60 (0.49–0.75), and 0.56 (0.44–0.73) in the Q2, Q3, and Q4 groups, respectively, compared to the Q1 group.

When analyzed by sex, the male and female results were consistent with the main analysis findings. In females, the HR (95% CI) for the Q2, Q3, and Q4 groups compared to the Q1 group was 0.63 (0.53–0.75), 0.60 (0.50–0.73), and 0.59 (0.47–0.75), respectively. In males, the HR (95% CI) for the Q2, Q3, and Q4 groups compared to the Q1 group was 0.58 (0.49–0.68), 0.58 (0.49–0.69), and 0.45 (0.37–0.55), respectively.

When stratifying by the BMI, only the group with a BMI <18.5 kg/m^2^ did not yield statistically significant results. This might have been due to the small sample size of this subgroup (*n*= 263). In the group with a BMI ≥18.5 kg/m^2^ and <25 kg/m^2^, the HR (95% CI) for the Q2, Q3, and Q4 groups compared to the Q1 group was 0.50 (0.40–0.62), 0.47 (0.38–0.59), and 0.37 (0.29–0.49), respectively. In the group with a BMI ≥25 kg/m^2^ and <30 kg/m^2^, the HR (95% CI) for the Q2, Q3, and Q4 groups was 0.62 (0.51–0.74), 0.61 (0.50–0.74), and 0.50 (0.40–0.64), respectively. In the group with a BMI ≥30 kg/m^2^, the HR (95% CI) for the Q2, Q3, and Q4 groups was 0.72 (0.57–0.90), 0.76 (0.59–0.97), and 0.68 (0.51–0.92), respectively.

Furthermore, no statistically significant differences were observed between the subgroups.

### 3.5. Sensitivity Analysis

After excluding individuals diagnosed within the first two years of follow-up, the association between a higher dietary iron intake and a lower risk of mental and behavioral disorders due to the use of tobacco remained unchanged (Supplementary Table S2). In Model 4, groups Q2 (HR: 0.60, 95% CI: 0.53–0.68, *p* < 0.001), Q3 (HR: 0.59, 95% CI: 0.52–0.67, *p* < 0.001), and Q4 (HR: 0.52, 95% CI: 0.44–0.61, *p* < 0.001) had reduced risk compared to group Q1.

Adjusting for sleep duration did not significantly change the main analysis results (Supplementary Table S3). In Model 4, compared to group Q1, the risk was reduced in the Q2 (HR: 0.61, 95% CI: 0.54–0.68, *p* < 0.001), Q3 (HR: 0.60, 95% CI: 0.53–0.67, *p* < 0.001), and Q4 (HR: 0.51, 95% CI: 0.43–0.59, *p* < 0.001) groups.

After excluding participants with extreme dietary iron intake or extreme energy intake, the findings remained consistent with the main analysis (Supplementary Tables S4 and S5). When participants with extreme dietary iron intake were removed, in Model 4, groups Q2 (HR: 0.62, 95% CI: 0.55–0.70, *p* < 0.001), Q3 (HR: 0.60, 95% CI: 0.52–0.68, *p* < 0.001), and Q4 (HR: 0.49, 95% CI: 0.42–0.57, *p* < 0.001) had reduced risk compared to group Q1. When participants with extreme energy intake were removed, in Model 4, groups Q2 (HR: 0.59, 95% CI: 0.53–0.67, *p* < 0.001), Q3 (HR: 0.57, 95% CI: 0.50–0.65, *p* < 0.001), and Q4 (HR: 0.48, 95% CI: 0.41–0.56, *p* < 0.001) had reduced risk compared to group Q1.

Finally, excluding participants with hypertension, stroke, and diabetes did not alter the association (Supplementary Table S6). In Model 3, compared to group Q1, the risk was reduced in the Q2 (HR: 0.59, 95% CI: 0.51–0.68, *p* < 0.001), Q3 (HR: 0.56, 95% CI: 0.48–0.65, *p* < 0.001), and Q4 (HR: 0.47, 95% CI: 0.39–0.56, *p* < 0.001) groups.

After additional adjustment for iron supplementation, the results were consistent with the main analysis (Supplementary Table S7). In Model 4, compared to group Q1, the risk was reduced in the Q2 (HR: 0.60, 95% CI: 0.53–0.68, *p* < 0.001), Q3 (HR: 0.59, 95% CI: 0.52–0.67, *p* < 0.001), and Q4 (HR: 0.50, 95% CI: 0.43–0.58, *p* < 0.001) groups.

In the analysis of heme iron, the cross-sectional analyses showed that dietary heme iron intake was not associated with the risk of mental and behavioral disorders due to the use of tobacco (Supplementary Table S8). The results of the longitudinal analyses indicated that they were consistent with the results of the cross-sectional analyses, except for the Q2 group in Model 1, which showed statistically significant results (HR: 0.86, 95% CI: 0.76–0.97, *p* = 0.012) (Supplementary Table S8). In the non-heme iron analyses, the results of the cross-sectional analyses indicated that dietary heme iron intake was associated with a reduced risk of mental and behavioral disorders due to the use of tobacco (Supplementary Table S8). The results of Model 4 showed that the OR (95% CI) was 0.42 (0.21–0.82), 0.31 (0.14–0.67), and 0.35 (0.15–0.84) for the Q2, Q3, and Q4 groups, respectively, compared to the Q1 group. The results of the longitudinal analyses supported the results of the cross-sectional analyses (Supplementary Table S8). The results of Model 4 showed that the HR (95% CI) for groups Q2, Q3, and Q4 was 0.59 (0.52–0.67), 0.57 (0.51–0.65), and 0.48 (0.42–0.56), respectively, compared to group Q1.

These sensitivity analysis results confirmed the robustness of the study findings.

### 3.6. Restricted Cubic Spline

**Figure 2:**
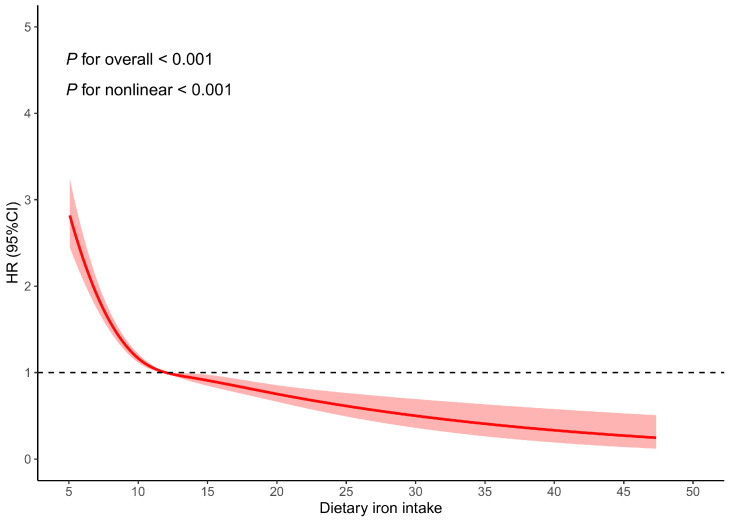
Restricted cubic spline plots of dietary iron intake and mental and behavioral disorders due to use of tobacco. Note: The red lines are HR estimates and the pink areas are 95%CI for HR.

The results of the restricted cubic spline analysis indicated a similar L-shaped nonlinear relationship between dietary iron intake and mental and behavioral disorders due to the use of tobacco. As dietary iron intake increased, the risk of these disorders decreased. A significant risk reduction was observed when the intake was approximately between 0 and 12 mg/day. However, the risk reduction rate slowed when the intake exceeded about 12 mg/day (Figure 2).

When stratified by age, the trend in risk changes was similar to what was previously described. The inflection point for the ≤60 years and the >60 years groups was around 12 mg/day (Supplementary Figure S1a,b).

When stratified by sex, the trend in risk change for males followed a similar pattern to that previously described, with an inflection point at approximately 13 mg/day (Supplementary Figure S1c). In females, however, an L-shape was observed between dietary iron intake and mental and behavioral disorders due to the use of tobacco. A significant risk reduction occurred when the intake was around 0 to 11 mg/day. When the intake exceeded approximately 11 mg/day, the risk reduction rate began to plateau (Supplementary Figure S1d).

## 4. Discussion

In this study, we conducted logistic regression and Cox regression analyses based on the UKB population sample, finding that a higher dietary iron intake was associated with a lower risk of mental and behavioral disorders due to the use of tobacco. The stratified analysis results supported the protective effect of dietary iron intake on mental and behavioral disorders due to the use of tobacco. The sensitivity analysis results were consistent with the main analysis results. Additionally, the restricted cubic spline analysis revealed a nonlinear relationship between dietary iron intake and mental and behavioral disorders due to the use of tobacco. In the overall population, both age groups, and the male group, the risk of increasing dietary iron intake initially decreased rapidly before slowing down. For females, the trend in risk change was characterized by a rapid initial decline followed by stabilization.

In this study, we observed that higher dietary iron intake was associated with a reduced risk of mental and behavioral disorders due to the use of tobacco. Although there is no epidemiological evidence linking dietary iron intake to mental and behavioral disorders due to the use of tobacco, a large number of previous studies have explored the relationship between iron and mental and behavioral disorders. It is evident that mental and behavioral disorders due to the use of tobacco are a subset of this broader category. For example, one clinical study found that iron deficiency significantly increased the risk of both psychiatric comorbidity and individual psychiatric disorders [39]. The results from another study supported the association of iron deficiency with depressive symptoms [40]. A clinical study revealed that low iron levels were associated with a significantly higher risk of developing mental disorders [41]. In addition, animal experiments found that rats fed an iron-deficient diet exhibited more anxiety-like behaviors than control rats, supporting the findings of observational studies in humans [42]. As mentioned above, these findings supported our results to some extent.

Mental and behavioral disorders due to the use of tobacco are primarily driven by nicotine dependence [43]. Nicotine is a sympathomimetic stimulant that binds to nicotinic acetylcholine receptors in the brain [44,45]. It releases dopamine and other neurotransmitters, such as norepinephrine, which induce feelings of pleasure [46]. Repeated exposure to nicotine increases the number of nicotinic acetylcholine receptors, altering the brain’s reward system and raising the threshold for pleasure [46,47]. When these receptors are not bound to nicotine, individuals may experience mental and behavioral disorders such as inattention, restlessness, and irritability [43]. Iron plays a crucial role in the synthesis and release of dopamine. On the one hand, iron can increase dopamine release by promoting the work of dopamine transporter proteins [48]. On the other hand, iron can enhance dopaminergic signaling by increasing the affinity and expression of D2 neurotransmitters [49]. In addition, iron is an essential cofactor for tyrosine hydroxylase, the rate-limiting enzyme in dopamine synthesis [50]. Sufficient iron levels can enhance tyrosine hydroxylase activity, thereby increasing dopamine production and alleviating mental and behavioral disorders [51].

This study had several strengths. Firstly, it represented the first exploration of the association between dietary iron intake and mental and behavioral disorders due to the use of tobacco. In addition, we explored the L-shaped and similar L-shape association between dietary iron intake and mental and behavioral disorders due to the use of tobacco using restricted cubic spline plots. Secondly, we combined both cross-sectional and longitudinal studies, verifying the results from the cross-sectional analysis with cohort studies to ensure reliability. Thirdly, we utilized a large population cohort from the UKB, enhancing the representativeness of our sample. Finally, we comprehensively included various covariates and conducted multiple sensitivity and stratified analyses to assess the robustness of our findings.

We acknowledged several limitations. Firstly, dietary iron intake and alcohol information was obtained from participants’ online questionnaires, which might introduce recall bias. Secondly, the prolonged data collection period of the UKB prevented us from distinguishing the effects of short-term versus long-term dietary iron intake on mental and behavioral disorders due to the use of tobacco. Thirdly, the study was ultimately observational, preventing us from drawing causal conclusions. Lastly, although we had attempted to adjust for numerous confounding factors, the influence of unknown factors may persist.

## 5. Conclusions

In conclusion, we found a stable, protective effect of dietary iron intake against mental and behavioral disorders due to the use of tobacco and a nonlinear relationship between dietary iron intake and mental and behavioral disorders due to the use of tobacco. Our findings may provide valuable insights into how individuals can reduce mental and behavioral disorders due to the use of tobacco by appropriately increasing dietary iron intake. Meanwhile, it offers reliable clues for future public health policy development.

## Figures and Tables

**Table 1:** Baseline characteristics of the quartile of dietary iron intake.

Characteristic	Dietary Iron Intake (mg/day)				*p* for Groups
	Q1 ≤9.35	Q2 >9.35 and ≤12.04	Q3 >12.04 and ≤15.10	Q4 >15.10	
Age (mean (SD))	55.79 (8.24)	56.55 (8.05)	56.70 (8.10)	56.58 (8.23)	<0.001
Sex (N (%))					<0.001
Female	8183 (29.66%)	7468 (27.07%)	6712 (24.33%)	5223 (18.93%)	
Male	4548 (19.49%)	5262 (22.55%)	6018 (25.79%)	7507 (32.17%)	
Ethnicity (N (%))					<0.001
White	11,527 (24.05%)	12,089 (25.23%)	12,182 (25.42%)	12,125 (25.3%)	
Other	1204 (40.16%)	641 (21.38%)	548 (18.28%)	605 (20.18%)	<0.001
BMI (mean (SD))	27.50 (5.00)	27.03 (4.64)	27.03 (4.61)	27.17 (4.66)	
Physical activity level (N (%))					<0.001
Low	2550 (29.16%)	2229 (25.49%)	2052 (23.46%)	1915 (21.90%)	
Moderate	5301 (25.20%)	5360 (25.48%)	5352 (25.44%)	5021 (23.87%)	
High	4880 (23.08%)	5141 (24.32%)	5326 (25.19%)	5794 (27.41%)	
Energy intake (mean (SD))					
All	6085.99 (1763.05)	7872.04 (1729.02)	9136.24 (1931.98)	11,640.68 (3042.33)	<0.001
Female	5926.63 (1686.01)	7610.64 (1643.24)	8728.24 (1794.75)	10,848.70 (2695.20)	<0.001
Male	6372.72 (1859.90)	8243.04 (1779.31)	9591.29 (1977.57)	12,191.71 (3146.82)	<0.001
TDI (mean (SD))	−1.04 (2.94)	−1.44 (2.74)	−1.47 (2.74)	−1.34 (2.79)	<0.001
Education qualifications (N (%))					<0.001
College and above	4980 (21.87%)	5672 (24.91%)	5917 (25.98%)	6203 (27.24%)	
Below College	7751 (27.54%)	7058 (25.07%)	6813 (24.20%)	6527 (23.19%)	
Alcohol consumption (N (%))					
All					<0.001
Never	658 (36.04%)	438 (23.99%)	400 (21.91%)	330 (18.07%)	
Previous	594 (33.07%)	424 (23.61%)	385 (21.44%)	393 (21.88%)	
Current	11,479 (24.27%)	11,868 (25.09%)	11,945 (25.25%)	12,007 (25.39%)	
Female					<0.001
Never	498 (37.67%)	313 (23.68%)	287 (21.71%)	224 (16.94%)	
Previous	350 (35.07%)	245 (24.55%)	221 (22.14%)	182 (18.24%)	
Current	7335 (29.03%)	6910 (27.35%)	6204 (24.55%)	4817 (19.07%)	
Male					<0.001
Never	160 (31.75%)	125 (24.80%)	113 (22.42%)	106 (21.03%)	
Previous	244 (30.58%)	179 (22.43%)	164 (20.55%)	211 (26.44%)	
Current	4144 (18.81%)	4958 (22.50%)	5741 (26.06%)	7190 (32.63%)	
Employment status (N (%))					<0.001
Full-time work	7610 (25.75%)	7445 (25.19%)	7268 (24.59%)	7232 (24.47%)	
Non-full-time work	5121 (23.97%)	5285 (24.74%)	5462 (25.56%)	5498 (25.73%)	
Hypertension (N (%))					0.083
Yes	3662 (24.92%)	3621 (24.64%)	3628 (24.69%)	3784 (25.75%)	
No	9069 (25.03%)	9109 (25.14%)	9102 (25.13%)	8946 (24.69%)	
Stroke (N (%))					0.685
Yes	266 (25.29%)	252 (23.95%)	256 (24.33%)	278 (26.43%)	
No	12,465 (25.00%)	12,478 (25.02%)	12,474 (25.01%)	12,452 (24.97%)	
Diabetes (N (%))					0.040
Yes	685 (26.13%)	641 (24.45%)	604 (23.04%)	692 (26.39%)	
No	12,046 (24.94%)	12,089 (25.03%)	12,126 (25.11%)	12,038 (24.92%)	<0.001

Note: N: numbers, SD: standard deviation, BMI: body mass index, TDI: Townsend deprivation index.

**Table 2:** Cross-sectional association between dietary iron intake and mental and behavioral disorders due to use of tobacco.

Model	Q2		Q3		Q4	
	OR (95% CI)	*p*-Value	OR (95% CI)	*p*-Value	OR (95% CI)	*p*-Value
Model 1	0.42 (0.22–0.80)	**0.008**	0.29 (0.14–0.61)	**0.001**	0.55 (0.30–0.99)	**0.046**
Model 2	0.44 (0.23–0.85)	**0.014**	0.29 (0.14–0.62)	**0.001**	0.51 (0.28–0.94)	**0.03** **0**
Model 3	0.42 (0.21–0.83)	**0.013**	0.27 (0.12–0.61)	**0.002**	0.43 (0.18–1.01)	0.053
Model 4	0.42 (0.21–0.83)	**0.012**	0.26 (0.12–0.60)	**0.001**	0.41 (0.18–0.98)	**0.044**

Note: Q1 was the reference group. Model 1 was not adjusted for covariates. Model 2 adjusted for age, sex, ethnicity, education qualifications, employment status, and TDI. Model 3 further adjusted for BMI, physical activity level, energy intake, and alcohol consumption. Model 4 further adjusted for hypertension, diabetes, and stroke. OR: odds ratio; CI: confidence interval. The *p*-values of significant results are shown in bold.

**Table 3:** Longitudinal association between dietary iron intake and mental and behavioral disorders due to use of tobacco.

Model	Q2			Q3			Q4		
	HR (95% CI)	*p*-Value	RR (95% CI)	HR (95% CI)	*p*-Value	RR (95% CI)	HR (95% CI)	*p*-Value	RR (95% CI)
Model 1	0.60 (0.53–0.68)	**<0.001**	0.64 (0.57–0.72)	0.59 (0.52–0.67)	**<0.001**	0.69 (0.61–0.77)	0.50 (0.43–0.59)	**<0.001**	0.74 (0.66–0.83)
Model 2	0.67 (0.59–0.75)	**<0.001**		0.70 (0.63–0.79)	**<0.001**		0.71 (0.63–0.79)	**<0.001**	
Model 3	0.60 (0.53–0.68)	**<0.001**		0.59 (0.52–0.67)	**<0.001**		0.50 (0.43–0.59)	**<0.001**	
Model 4	0.60 (0.53–0.68)	**<0.001**		0.59 (0.52–0.67)	**<0.001**		0.50 (0.43–0.58)	**<0.001**	

Note: Q1 was the reference group. Model 1 was not adjusted for covariates. Model 2 adjusted for age, sex, ethnicity, education qualifications, employment status, and TDI. Model 3 further adjusted for BMI, physical activity level, energy intake, and alcohol consumption. Model 4 further adjusted for hypertension, diabetes, and stroke. HR: hazard ratio; RR: risk ratio; CI: confidence interval. The *p*-values of significant results are shown in bold.

## Data Availability

The data supporting the findings of this study are publicly available at UK Biobank (https://www.ukbiobank.ac.uk, accessed on 1 December 2024).

## Supplementary Materials

The following supporting information can be downloaded at: https://www.mdpi.com/article/10.3390/nu17010039/s1, Figure S1: Restricted cubic spline plots of dietary iron intake and mental and behavioral disorders due to use of tobacco stratified by sex or age; Table S1: Stratified analysis of associations between dietary iron intake and mental and behavioral disorders due to use of tobacco based on baseline age, sex, and BMI (Model 4); Table S2: Longitudinal associations between dietary iron intake and mental and behavioral disorders due to use of tobacco after excluding participants with the onset of disease in the first two years of follow-up; Table S3: Longitudinal associations between dietary iron intake and mental and behavioral disorders due to use of tobacco after correcting for sleep duration effects; Table S4: Longitudinal associations between dietary iron intake and mental and behavioral disorders due to use of tobacco after excluding participants with extreme dietary iron intake; Table S5: Longitudinal associations between dietary iron intake and mental and behavioral disorders due to use of tobacco after excluding participants with extreme energy intake; Table S6: Longitudinal associations between dietary iron intake and mental and behavioral disorders due to use of tobacco after excluding participants with hypertension, stroke, and diabetes; Table S7: Longitudinal associations between dietary iron intake and mental and behavioral disorders due to use of tobacco after adjustment for iron supplements; Table S8: Association analysis between different types of dietary iron intake and mental and behavioral disorders due to use of tobacco after categorization of dietary iron intake.
