# Supplementary material for: Dietary Iron Intake and Mental and Behavioral Disorders Due to Use of Tobacco: A UK Biobank Study
Source: Nutrients. 2024 Dec 26;17(1):39. doi: 10.3390/nu17010039 (PMC11722800; doi:10.3390/nu17010039)
Supplement: Supplementary file 1 [file nutrients-17-00039-s001.zip › nutrients-3378414-supplementary.pdf]

Supplementary material

Dietary Iron Intake and Mental and Behavioral Disorders Due to Use of Tobacco: A UK Biobank Study

Xueting Qi, Ronghui Zhang, Hailong Zhu, Jia Luo, Qiuge Zhang, Weijing Wang, Tong Wang and Dongfeng Zhang

Supplementary material Contents

Supplementary Table S1 Stratified analysis of associations between dietary iron intake and mental and behavioral disorders due to use of tobacco based on baseline age, sex, and BMI (Model 4)..3

Supplementary Table S2 Longitudinal associations between dietary iron intake and mental and behavioral disorders due to use of tobacco after excluding participants with the onset of disease in the first two years of follow-up. 3

Supplementary Table S3 Longitudinal associations between dietary iron intake and mental and behavioral disorders due to use of tobacco after correcting for sleep duration effects. 4

Supplementary Table S4 Longitudinal associations between dietary iron intake and mental and behavioral disorders due to use of tobacco after excluding participants with extreme dietary iron intake..4

Supplementary Table S5 Longitudinal associations between dietary iron intake and mental and behavioral disorders due to use of tobacco after excluding participants with extreme energy intake..4

Supplementary Table S6 Longitudinal associations between dietary iron intake and mental and behavioral disorders due to use of tobacco after excluding participants with hypertension, stroke, and diabetes..5

Supplementary Table S7 Longitudinal associations between dietary iron intake and mental and behavioral disorders due to use of tobacco after adjustment for iron supplements. 5

Supplementary Table S8 Association analysis between different types of dietary iron intake and mental and behavioral disorders due to use of tobacco after categorization of dietary iron intake. 6

Supplementary Figure S1 Restricted cubic spline plots of dietary iron intake and mental and behavioral disorders due to use of tobacco stratified by sex or age..7

Supplementary Table S1 Stratified analysis of associations between dietary iron intake and mental and behavioral disorders due to use of tobacco based on baseline age, sex, and BMI (Model 4).

Characters	Q2				Q3				Q4			
	HR (95%CI)	*P*-value	*P*-value for interaction	RR (95%CI)	HR (95%CI)	*P*-value	*P*-value for interaction	RR (95%CI)	HR (95%CI)	*P*-value	*P*-value for interaction	RR (95%CI)
Age												
≤ 60 years	0.58 (0.50 - 0.67)	**<0.001**	0.612	0.63 (0.54 - 0.72)	0.57 (0.49 - 0.67)	**<0.001**	0.355	0.68 (0.59 - 0.78)	0.45 (0.37 - 0.55)	**<0.001**	0.060	0.70 (0.61 - 0.81)
> 60 years	0.62 (0.51 - 0.76)	**<0.001**		0.68 (0.56 - 0.83)	0.60 (0.49 - 0.75)	**<0.001**		0.72 (0.60 - 0.88)	0.56 (0.44 - 0.73)	**<0.001**		0.82 (0.68 - 0.99)
Sex												
Female	0.63 (0.53 - 0.75)	**<0.001**	0.638	0.64 (0.55 - 0.76)	0.60 (0.50 - 0.73)	**<0.001**	0.740	0.63 (0.53 - 0.75)	0.59 (0.47 - 0.75)	**<0.001**	0.247	0.70 (0.59 - 0.84)
Male	0.58 (0.49 - 0.68)	**<0.001**		0.61 (0.51 - 0.72)	0.58 (0.49 - 0.69)	**<0.001**		0.67 (0.57 - 0.78)	0.45 (0.37 - 0.55)	**<0.001**		0.64 (0.55 - 0.75)
BMI												
<18.5 kg/m^2	0.77 (0.22 - 2.63)	0.671	0.096	0.82 (0.25 - 2.69)	0.20 (0.02 - 1.95)	0.167	0.228	0.22 (0.03 - 1.85)	0.87 (0.16 - 4.57)	0.866	0.351	1.33 (0.44 - 4.08)
≥18.5 kg/m^2 & <25 kg/m^2	0.50 (0.40 - 0.62)	**<0.001**		0.58 (0.47 - 0.72)	0.47 (0.38 - 0.59)	**<0.001**		0.64 (0.52 - 0.79)	0.37 (0.29 - 0.49)	**<0.001**		0.76 (0.62 - 0.93)
≥25 kg/m^2 & <30 kg/m^2	0.62 (0.51 - 0.74)	**<0.001**		0.65 (0.54 - 0.78)	0.61 (0.50 - 0.74)	**<0.001**		0.69 (0.58 - 0.82)	0.50 (0.40 - 0.64)	**<0.001**		0.70 (0.59 - 0.84)
≥30 kg/m^2	0.72 (0.57 - 0.90)	**0.005**		0.72 (0.57 - 0.90)	0.76 (0.59 - 0.97)	**0.028**		0.79 (0.63 - 0.98)	0.68 (0.51 - 0.92)	**0.013**		0.76 (0.61 - 0.95)

Note: BMI: Body mass index. Model 4 adjusted for age, sex, ethnicity, education qualifications, employment status, TDI, BMI, physical activity level, energy intake, alcohol consumption, hypertension, diabetes, and stroke.

Q1 was the reference group.

HR: hazard ratio; RR: risk ratio; CI: confidence interval.

The *P*-values of significant results are shown in bold.

Supplementary Table S2 Longitudinal associations between dietary iron intake and mental and behavioral disorders due to use of tobacco after excluding participants with the onset of disease in the first two years of follow-up.

Model	Q2			Q3			Q4		
	HR (95%CI)	*P*-value	RR (95%CI)	HR (95%CI)	*P*-value	RR (95%CI)	HR (95%CI)	*P*-value	RR (95%CI)
Model1	0.63 (0.56 - 0.72)	**<0.001**		0.68 (0.61 - 0.77)	**<0.001**		0.75 (0.67 - 0.85)	**<0.001**	
Model2	0.66 (0.59 - 0.75)	**<0.001**	0.64	0.70 (0.62 - 0.79)	**<0.001**	0.69	0.73 (0.65 - 0.82)	**<0.001**	0.76
Model3	0.60 (0.53 - 0.68)	**<0.001**	(0.57 - 0.72)	0.59 (0.52 - 0.67)	**<0.001**	(0.61 - 0.78)	0.52 (0.44 - 0.61)	**<0.001**	(0.67 - 0.86)
Model4	0.60 (0.53 - 0.68)	**<0.001**		0.59 (0.52 - 0.67)	**<0.001**		0.52 (0.44 - 0.61)	**<0.001**	

Note: Q1 was the reference group. Model 1 was not adjusted for covariates. Model 2 adjusted for age, sex, ethnicity, education qualifications, employment status, and TDI. Model 3 further adjusted for BMI, physical activity level, energy intake, and alcohol consumption.

HR: hazard ratio; RR: risk ratio; CI: confidence interval.

The *P*-values of significant results are shown in bold.

Supplementary Table S3 Longitudinal associations between dietary iron intake and mental and behavioral disorders due to use of tobacco after correcting for sleep duration effects.

Model	Q2			Q3			Q4		
	HR (95%CI)	*P*-value	RR (95%CI)	HR (95%CI)	*P*-value	RR (95%CI)	HR (95%CI)	*P*-value	RR (95%CI)
Model1	0.64 (0.57 - 0.72)	**<0.001**	0.64 (0.57 - 0.72)	0.69 (0.62 - 0.78)	**<0.001**	0.69 (0.61 - 0.77)	0.74 (0.66 - 0.82)	**<0.001**	0.74 (0.66 - 0.83)
Model2	0.68 (0.60 - 0.76)	**<0.001**		0.71 (0.64 - 0.80)	**<0.001**		0.71 (0.64 - 0.80)	**<0.001**	
Model3	0.61 (0.54 - 0.69)	**<0.001**		0.60 (0.53 - 0.68)	**<0.001**		0.51 (0.44 - 0.59)	**<0.001**	
Model4	0.61 (0.54 - 0.68)	**<0.001**		0.60 (0.53 - 0.67)	**<0.001**		0.51 (0.43 - 0.59)	**<0.001**	

Note: Q1 was the reference group. Model 1 adjusted for sleep duration. Model 2 further adjusted for age, sex, ethnicity, education qualifications, employment status, TDI, and sleep duration. Model 3 further adjusted for BMI, physical activity level, energy intake, alcohol consumption, and sleep duration. Model 4 further adjusted for hypertension, diabetes, stroke, and sleep duration.

HR: hazard ratio; RR: risk ratio; CI: confidence interval.

The *P*-values of significant results are shown in bold.

Supplementary Table S4 Longitudinal associations between dietary iron intake and mental and behavioral disorders due to use of tobacco after excluding participants with extreme dietary iron intake.

Model	Q2			Q3			Q4		
	HR (95%CI)	*P*-value	RR (95%CI)	HR (95%CI)	*P*-value	RR (95%CI)	HR (95%CI)	*P*-value	RR (95%CI)
Model1	0.67 (0.60 - 0.75)	**<0.001**	0.68 (0.60 - 0.76)	0.72 (0.64 - 0.81)	**<0.001**	0.73 (0.65 - 0.82)	0.75 (0.67 - 0.84)	**<0.001**	0.75 (0.67 - 0.85)
Model2	0.70 (0.62 - 0.79)	**<0.001**		0.74 (0.66 - 0.83)	**<0.001**		0.72 (0.64 - 0.81)	**<0.001**	
Model3	0.62 (0.55 - 0.70)	**<0.001**		0.60 (0.53 - 0.68)	**<0.001**		0.49 (0.42 - 0.58)	**<0.001**	
Model4	0.62 (0.55 - 0.70)	**<0.001**		0.60 (0.52 - 0.68)	**<0.001**		0.49 (0.42 - 0.57)	**<0.001**	

Note: Q1 was the reference group. Model 1 was not adjusted for covariates. Model 2 adjusted for age, sex, ethnicity, education qualifications, employment status, and TDI. Model 3 further adjusted for BMI, physical activity level, energy intake, and alcohol consumption.

HR: hazard ratio; RR: risk ratio; CI: confidence interval.

The *P*-values of significant results are shown in bold.

Supplementary Table S5 Longitudinal associations between dietary iron intake and mental and behavioral disorders due to use of tobacco after excluding participants with extreme energy intake.

Model	Q2			Q3			Q4		
	HR (95%CI)	*P*-value	RR (95%CI)	HR (95%CI)	*P*-value	RR (95%CI)	HR (95%CI)	*P*-value	RR (95%CI)
Model1	0.64 (0.57 - 0.72)	**<0.001**	0.65 (0.57 - 0.73)	0.69 (0.61 - 0.77)	**<0.001**	0.69 (0.62 - 0.78)	0.71 (0.63 - 0.79)	**<0.001**	0.74 (0.64 - 0.80)
Model2	0.67 (0.60 - 0.75)	**<0.001**		0.71 (0.63 - 0.79)	**<0.001**		0.69 (0.62 - 0.78)	**<0.001**	
Model3	0.60 (0.53 - 0.67)	**<0.001**		0.57 (0.50 - 0.66)	**<0.001**		0.48 (0.41 - 0.56)	**<0.001**	

Model	Q2			Q3			Q4		
	HR (95%CI)	*P*-value	RR (95%CI)	HR (95%CI)	*P*-value	RR (95%CI)	HR (95%CI)	*P*-value	RR (95%CI)
Model4	0.59 (0.53 - 0.67)	**<0.001**		0.57 (0.50 - 0.65)	**<0.001**		0.48 (0.41 - 0.56)	**<0.001**	

Note: Q1 was the reference group. Model 1 was not adjusted for covariates. Model 2 adjusted for age, sex, ethnicity, education qualifications, employment status, and TDI. Model 3 further adjusted for BMI, physical activity level, energy intake, and alcohol consumption.

HR: hazard ratio; RR: risk ratio; CI: confidence interval.

The *P*-values of significant results are shown in bold.

Supplementary Table S6 Longitudinal associations between dietary iron intake and mental and behavioral disorders due to use of tobacco after excluding participants with hypertension, stroke, and diabetes.

Model	Q2			Q3			Q4		
	HR (95%CI)	*P*-value	RR (95%CI)	HR (95%CI)	*P*-value	RR (95%CI)	HR (95%CI)	*P*-value	RR (95%CI)
Model1	0.64 (0.55 - 0.73)	**<0.001**		0.67 (0.58 - 0.77)	**<0.001**		0.74 (0.65 - 0.85)	**<0.001**	
Model2	0.67 (0.58 - 0.77)	**<0.001**	0.64 (0.56 - 0.74)	0.69 (0.60 - 0.80)	**<0.001**	0.67 (0.58 - 0.78)	0.71 (0.62 - 0.82)	**<0.001**	0.75 (0.65 - 0.86)
Model3	0.59 (0.51 - 0.68)	**<0.001**		0.56 (0.48 - 0.65)	**<0.001**		0.47 (0.39 - 0.56)	**<0.001**	

Note: Q1 was the reference group. Model 1 was not adjusted for covariates. Model 2 adjusted for age, sex, ethnicity, education qualifications, employment status, and TDI. Model 3 further adjusted for BMI, physical activity level, energy intake, and alcohol consumption.

HR: hazard ratio; RR: risk ratio; CI: confidence interval.

The *P*-values of significant results are shown in bold.

Supplementary Table S7 Longitudinal associations between dietary iron intake and mental and behavioral disorders due to use of tobacco after adjustment for iron supplements.

Model	Q2			Q3			Q4		
	HR (95%CI)	*P*-value	RR (95%CI)	HR (95%CI)	*P*-value	RR (95%CI)	HR (95%CI)	*P*-value	RR (95%CI)
Model1	0.63 (0.57- 0.71)	**<0.001**		0.68 (0.61- 0.76)	**<0.001**		0.73 (0.65- 0.82)	**<0.001**	
Model2	0.67 (0.59- 0.75)	**<0.001**	0.64 (0.57 - 0.72)	0.70 (0.63- 0.79)	**<0.001**	0.69 (0.61 - 0.77)	0.71 (0.63- 0.79)	**<0.001**	0.74 (0.66 - 0.83)
Model3	0.60 (0.53- 0.68)	**<0.001**		0.59 (0.52- 0.67)	**<0.001**		0.50 (0.43- 0.59)	**<0.001**	
Model4	0.60 (0.53- 0.68)	**<0.001**		0.59 (0.52- 0.67)	**<0.001**		0.50 (0.43- 0.58)	**<0.001**	

Note: Q1 was the reference group. Model 1 adjusted for iron supplements. Model 2 further adjusted for age, sex, ethnicity, education qualifications, employment status, TDI, and iron supplements. Model 3 further adjusted for BMI, physical activity level, energy intake, alcohol consumption, and iron supplements. Model 4 further adjusted for hypertension, diabetes, stroke, and iron supplements.

HR: hazard ratio; RR: risk ratio; CI: confidence interval.

The *P*-values of significant results are shown in bold.

Supplementary Table S8 Association analysis between different types of dietary iron intake and mental and behavioral disorders due to use of tobacco after categorization of dietary iron intake.

Model	Q2			Q3			Q4		
	OR or HR (95%CI)	*P*-value	RR (95%CI)	OR or HR (95%CI)	*P*-value	RR (95%CI)	OR or HR (95%CI)	*P*-value	RR (95%CI)
Cross-sectional analysis									
Heme iron									
Model1	0.65 (0.23 - 1.78)	0.202		0.73 (0.28 - 1.94)	0.344		0.83 (0.32 - 2.12)	0.546	
Model2	0.70 (0.25 - 1.93)	0.293		0.76 (0.29 - 2.03)	0.415		0.82 (0.32 - 2.12)	0.542	
Model3	0.71 (0.26 - 1.97)	0.323		0.80 (0.30 - 2.16)	0.509		0.89 (0.33 - 2.35)	0.711	
Model4	0.71 (0.25 - 1.96)	0.312		0.79 (0.29 - 2.13)	0.477		0.85 (0.32 - 2.25)	0.616	
Non-heme iron									
Model1	0.42 (0.22 - 0.80)	**<0.001**		0.35 (0.18 - 0.71)	**<0.001**		0.48 (0.26 - 0.90)	**<0.001**	
Model2	0.44 (0.23 - 0.85)	**<0.001**		0.36 (0.18 - 0.73)	**<0.001**		0.45 (0.24 - 0.85)	**<0.001**	
Model3	0.41 (0.21 - 0.81)	**<0.001**		0.32 (0.15 - 0.68)	**<0.001**		0.36 (0.15 - 0.85)	**<0.001**	
Model4	0.42 (0.21 - 0.82)	**<0.001**		0.31 (0.14 - 0.67)	**<0.001**		0.35 (0.15 - 0.84)	**<0.001**	
Longitudinal analysis									
Heme iron									
Model1	0.86 (0.76 - 0.97)	**0.012**		0.93 (0.83 - 1.04)	0.084		1.07 (0.96 - 1.19)	0.094	
Model2	0.90 (0.80 - 1.01)	0.084	0.86	0.94 (0.84 - 1.06)	0.326	0.93	1.05 (0.94 - 1.18)	0.371	1.07
Model3	0.90 (0.80 - 1.02)	0.094	(0.77 - 0.97)	0.94 (0.84 - 1.06)	0.290	(0.83 - 1.05)	1.04 (0.92 - 1.16)	0.541	(0.95 - 1.20)
Model4	0.91 (0.80 - 1.02)	0.101		0.94 (0.83 - 1.05)	0.284		1.03 (0.92 - 1.16)	0.585	
Non-heme iron									
Model1	0.63 (0.56 - 0.70)	**<0.001**		0.67 (0.60 - 0.75)	**<0.001**		0.71 (0.64 - 0.79)	**<0.001**	
Model2	0.66 (0.59 - 0.74)	**<0.001**	0.64	0.69 (0.62 - 0.78)	**<0.001**	0.68	0.69 (0.62 - 0.77)	**<0.001**	0.72
Model3	0.59 (0.53 - 0.67)	**<0.001**	(0.56 - 0.71)	0.58 (0.51 - 0.65)	**<0.001**	(0.60 - 0.76)	0.49 (0.42 - 0.57)	**<0.001**	(0.64 - 0.80)
Model4	0.59 (0.52 - 0.67)	**<0.001**		0.57 (0.51 - 0.65)	**<0.001**		0.48 (0.42 - 0.56)	**<0.001**	

Note: Q1 was the reference group. Model 1 was not adjusted for covariates. Model 2 adjusted for age, sex, ethnicity, education qualifications, employment status, and TDI. Model 3 further adjusted for BMI, physical activity level, energy intake, and alcohol consumption.

OR: odds ratio; HR: hazard ratio; RR: risk ratio; CI: confidence interval.

The *P*-values of significant results are shown in bold.

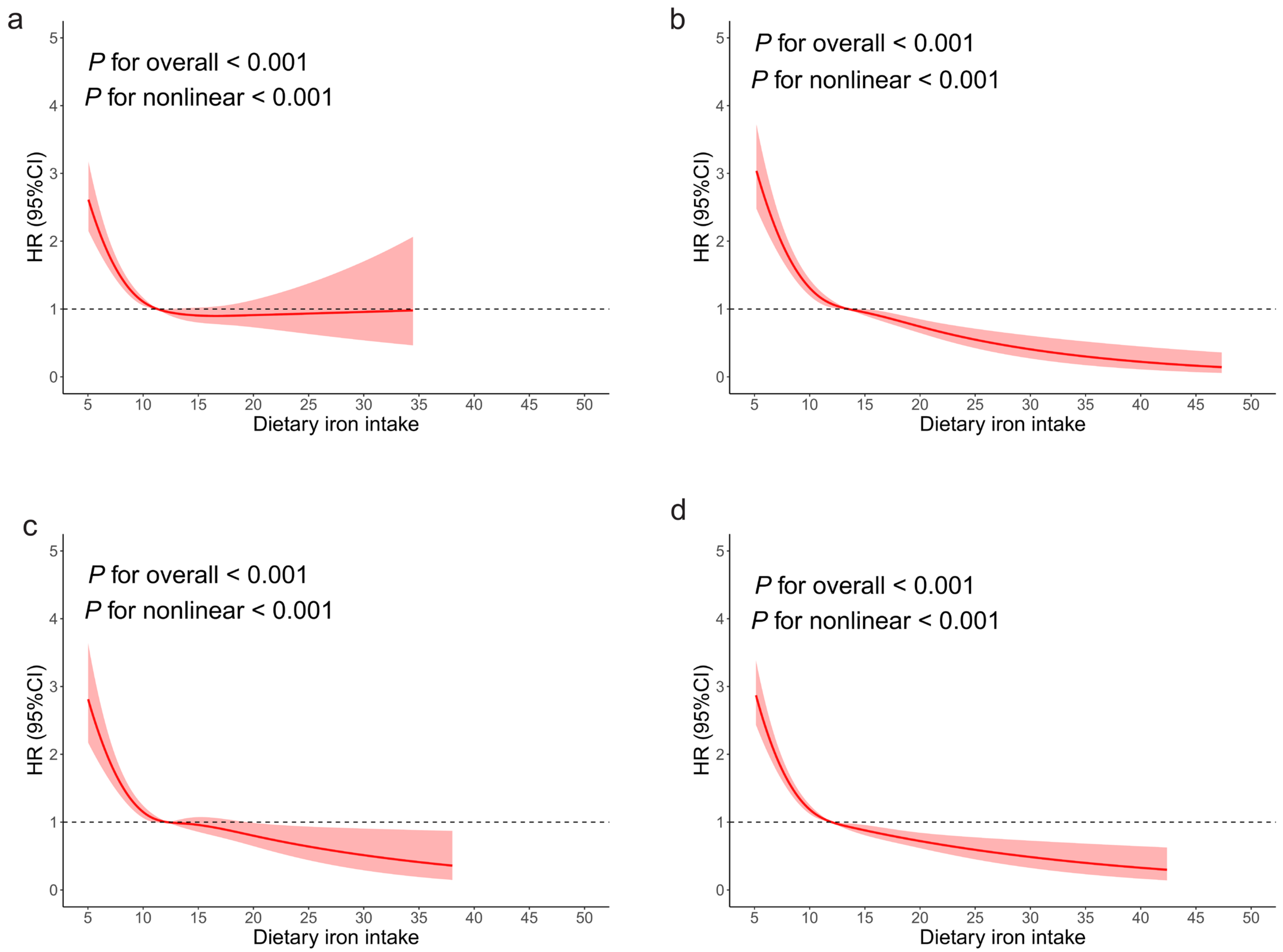

Supplementary Figure S1 Restricted cubic spline plots of dietary iron intake and mental and behavioral disorders due to use of tobacco stratified by sex or age.

Note: a: ≤ 60 years, b: > 60 years, c: female, d: male. The red lines are HR estimates and the pink areas are 95%CI for HR. HR: hazard ratios; CI: confidence intervals.
